# Growth performance and element concentrations reveal the calcicole-calcifuge behavior of three *Adiantum* species

**DOI:** 10.1186/s12870-020-02538-6

**Published:** 2020-07-10

**Authors:** Jian Xiong Liao, Dan Yang Liang, Qian Wen Jiang, Ling Mo, Gao Zhong Pu, Deng Zhang

**Affiliations:** 1 Guangxi Key Laboratory of Plant Conservation and Restoration Ecology in Karst Terrain, Guangxi Institute of Botany, Guangxi Zhuang Autonomous Region and Chinese Academy of Sciences, Guilin, P.R. China; 2 College of Tourism & Landscape Architecture (College of Plant and Ecological Engineering), Guilin University of Technology, Guilin, P.R. China

**Keywords:** *Adiantum* species, Acidic soils, Calcareous soils, Calcicole, Calcifuge, Element concentration

## Abstract

**Background:**

The calcicole or calcifuge behavior of wild plants has been related to element deficiency or toxicity. For fern species, however, knowledge about their adaptive differences and responses to soil environmental changes is virtually absent. In the karst regions of southern China, most *Adiantum* species favor calcareous soils, but *A. flabellulatum* prefers acidic soils. Such contrasting preferences for soil types in the same genus are interesting and risky because their preferred soils may “pollute” each other due to extreme precipitation events. We mixed calcareous and acidic soils at 1:1 (v/v) to simulate the “polluted” soils and grew three *Adiantum* species (the calcicole *A. capillus-veneris* f. *dissectum* and *A. malesianum* and the calcifuge *A. flabellulatum*) on the calcareous, acidic and mixed soils for 120 d and assessed their growth performance and element concentrations.

**Results:**

The calcareous soil showed the highest pH, Ca, Mg and P concentrations but the lowest K concentration, followed by the mixed soil, and the acidic soil. After 120 d of growth, the calcifuge *A. flabellulatum* on the calcareous and mixed soils exhibited lower SPAD and relative growth rate (RGR) than those on the acidic soil, and its leaf and root Ca, Mg and Fe concentrations were higher and K was lower on the calcareous soil than on the acidic soil. The calcicole *A. capillus-veneris* f. *dissectum* on the calcareous soil had similar leaf element concentrations and RGR with those on the mixed soil, but their leaf Ca, Fe and Al were lower and leaf P and K concentrations, SPAD and RGR were higher than those on the acidic soil. For the calcicole *A. malesianum*, leaf Ca, Fe and Al were lowest and leaf P and RGR were highest when grown on the mixed soil, intermediated on the calcareous soil, and on the acidic soil. Compared with *A. malesianum*, *A. capillus-veneris* f. *dissectum* had lower leaf Ca, Fe and Al but higher leaf Mg concentration when grown on the same calcareous or mixed soils.

**Conclusions:**

*A. capillus-veneris* f. *dissectum* is a low leaf Ca calcicole species while *A. malesianum* is an Al accumulating calcicole species. They can effectively take up P and K to leaves and hence can thrive on calcareous soils. In contrast, the calcifuge *A. flabellulatum* grown on calcareous soils is stunted. Such growth performance may be attributed to the increased leaf Ca and decreased leaf K concentration. If their preferred soils are “polluted”, *A. flabellulatum* can grow worse, *A. capillus-veneris* f. *dissectum* can remain almost unaffected while *A. malesianum* will perform better.

## Background

Calcareous and acidic soils are the predominant soil types in the karst regions of southern China [1, 2]. They are adjacent but strongly differ in their vegetation [3, 4]. With the increase of economic activities and extreme rainstorm frequencies, the two contrasting soils may “pollute” each other [5] and hence their specialized wild plants may face survival risk. Therefore, studies on the adaptive differences and mechanisms of these calcicole-calcifuge plants are crucial for understanding their growth and distribution and for predicting their responses to possible soil environmental changes.

Element deficiency or toxicity is thought to relate to calcicole or calcifuge behavior of wild plants. On calcareous soils, calcicole plants can regulate nutrient uptake via root architectural or exudation plasticity [6, 7], while calcifuge species are limited by low nutrient availability [6, 8] or deleterious precipitation of Ca-phosphate [9] or Ca-enhanced P toxicity [10]. In turn, calcicole plants can suffer from toxicity symptoms by excess Al^3+^ or Fe^2+^ at acidic sites [11, 12]. In these existing studies, most calcicole and/or calcifuge species were seed plants and they were generally grown on calcareous or acidic soils [9, 13]. For fern species, to the best of our knowledge, the mechanisms causing their calcicole-calcifuge behavior are largely unexplored. Moreover, plants grown on calcareous and acidic mixed soils, to analyze their responses to “polluted” soil conditions, are also virtually absent.

*Adiantum* species (Pteridaceae) are important medicinal, ornamental ferns and environmental indicators. In the karst regions of southern China, most of them are found on calcareous soils, but *A. flabellulatum* only grows on acidic soils [14]. Such contrasting preferences for soil types in the same genus are interesting, but are not well understood [15]. In this context, we selected three *Adiantum* species (the calcicole *A. capillus-veneris* f. *dissectum* and *A. malesianum*, and the calcifuge *A. flabellulatum*) and grew them on calcareous, acidic and mixed soils. We assessed their biomass, relative growth rate (RGR), chlorophyll (SPAD values) and element concentrations. The objectives were to compare the performance of three *Adiantum* species on three soil types. We hypothesized that: (i) on calcareous soils, the calcicole *A. capillus-veneris* f. *dissectum* and *A. malesianum* would perform better than the calcifuge *A. flabellulatum*, and that on acidic soils, the opposite would be true; (ii) all three species would perform worse on mixed soils than on their respective optimum soils; (iii) their calcicole or calcifuge behavior might be attributed to element deficiency and/or toxicity.

## Results

### Soil properties

The calcareous soil had higher pH, Ca, Mg and P concentrations but lower K concentration when compared with the acidic soil (*P* < 0.05, Table 1). After mixing the calcareous and acidic soils at 1:1, the mixed soil had similar pH with the calcareous soil (*P* > 0.05), but its Ca, Mg, P and K concentrations were intermediate between the calcareous and acidic soils (*P* < 0.05). For Fe and Al concentrations, the three soil types did not differ (*P* > 0.05).

**Table 1:** Chemical analysis of the acidic, calcareous and mixed soils used in this study

	Acidic soil	Calcareous soil	Mixed soil
pH (in CaCl_2_)	4.71 ± 0.14^b^	7.76 ± 0.04^a^	7.50 ± 0.04^a^
Ca (mg g^− 1^)	0.53 ± 0.03^c^	66.35 ± 1.13^a^	30.75 ± 1.04^b^
Mg (mg g^− 1^)	2.83 ± 0.04^c^	32.48 ± 0.53^a^	16.86 ± 0.57^b^
Fe (mg g^− 1^)	43.30 ± 1.05^a^	47.67 ± 1.26^a^	46.58 ± 1.40^a^
Al (mg g^− 1^)	69.71 ± 1.85^a^	76.26 ± 2.17^a^	73.52 ± 1.27^a^
P (mg g^− 1^)	0.37 ± 0.01^c^	1.00 ± 0.03^a^	0.73 ± 0.02^b^
K (mg g^−1^)	12.68 ± 0.09^a^	2.59 ± 0.09^c^	7.38 ± 0.25^b^

All the element concentrations are total concentrations. Values are means ± SE of 3 replicates from one-way ANOVA. Different letters indicate significant differences among soil types (*P* < 0.05).

### Plant growth characteristics

**Fig. 1:**
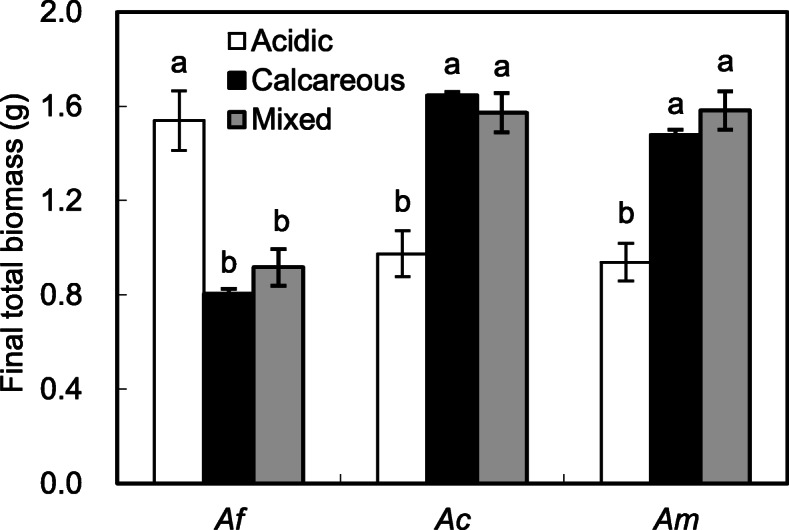
Final total biomass of the calcifuge *Adiantum flabellulatum* (*Af*) and the calcicole *A. capillus-veneris* f. *dissectum* (*Ac*) and *A. malesianum* (*Am*) grown on acidic, calcareous and mixed soils. Bar are means ± SE of 7 replicates from two-way ANCOVA. Different letters indicate significant differences in the same species (*P* < 0.05)

After 120 d of growth, *A. flabellulatum* on the calcareous and mixed soils were stunted and total biomass were lower than those grown on the acidic soil (*P* < 0.05, Fig. 1). In contrast, *A. capillus-veneris* f. *dissectum* and *A. malesianum* performed worse and total biomass were lower when grown on the acidic soil (*P* < 0.05). When grown on the mixed soil, all three species had similar total biomass with those grown on the calcareous soil (*P* > 0.05).

**Fig. 2:**
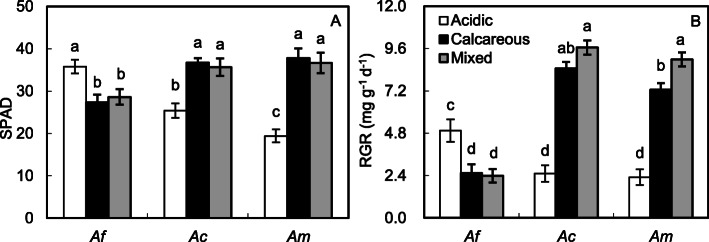
SPAD values (**a**) and relative growth rate (RGR, **b**) of the calcifuge *Adiantum flabellulatum* (*Af*) and the calcicole *A. capillus-veneris* f. *dissectum* (*Ac*) and *A. malesianum* (*Am*) grown on acidic, calcareous and mixed soils. Bar are means ± SE of 7 replicates from two-way ANOVA (**a**) or two-way ANCOVA (**b**). Different letters indicate significant differences across all species (*P* < 0.05)

When grown on the calcareous and mixed soils, *A. flabellulatum* exhibited chlorotic and necrotic spots on pinnules and lower chlorophyll contents (SPAD values) than those grown on the acidic soil (*P* < 0.05, Fig. 2a). Conversely, *A. capillus-veneris* f. *dissectum* and *A. malesianum* showed chlorotic symptoms and the lowest chlorophyll contents when grown on the acidic soil. For the symptoms, the margins of older pinnules of *A. capillus-veneris* f. *dissectum* were scorched while *A.malesianum* exhibited chlorotic stripes on older pinnules and then whole pinna shriveled. RGR of *A. flabellulatum* was higher when grown on the acidic soil than on the calcareous and mixed soils (*P* < 0.05, Fig. 2b). In *A. capillus-veneris* f. *dissectum* and *A. malesianum*, however, the highest and the lowest RGR occurred on the mixed and acidic soils, respectively. Compared with *A. flabellulatum*, *A. capillus-veneris* f. *dissectum* and *A. malesianum* had higher chlorophyll contents and RGR when grown on the calcareous or mixed soil (*P* < 0.05, Fig. 2). When grown on the acidic soil, chlorophyll contents and RGR of *A. capillus-veneris* f. *dissectum* and *A. malesianum* were lower than those of *A. flabellulatum* (*P* < 0.05).

### Plant element concentrations

**Fig. 3:**
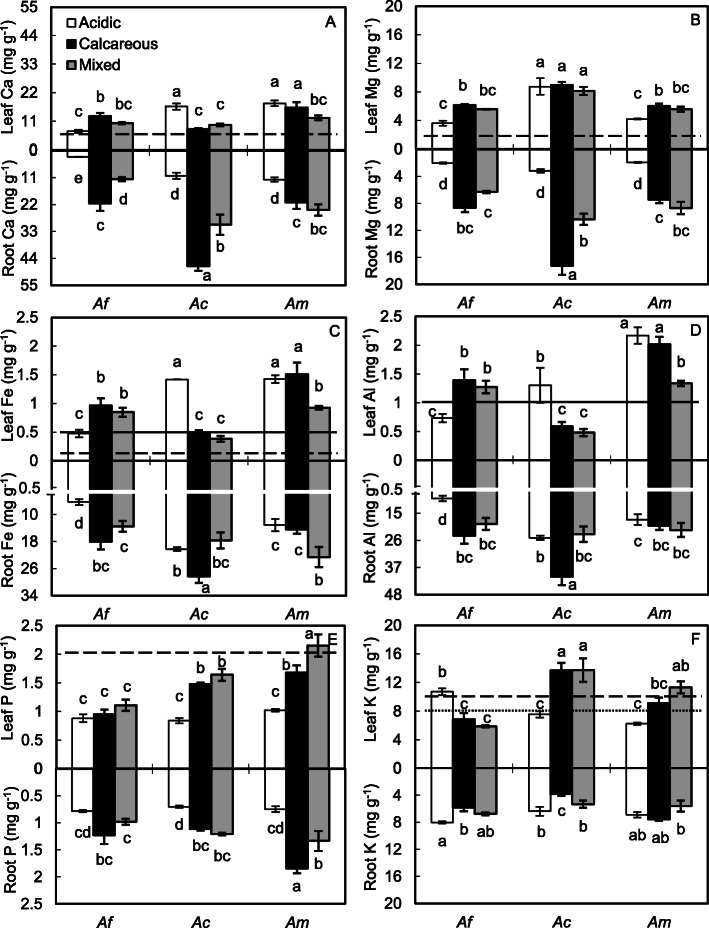
Element concentrations in leaves and roots of the calcifuge *Adiantum flabellulatum* (*Af*) and the calcicole *A. capillus-veneris* f. *dissectum* (*Ac*) and *A. malesianum* (*Am*) grown on acidic, calcareous and mixed soils. Bar are means ± SE of 7 replicates from two-way ANOVA. Different letters indicate significant differences within the same tissue and element (*P* < 0.05). The dashed lines represent the average leaf element concentrations sufficient for adequate growth [16]. The solid lines represent the critical Fe toxicity concentration [17] or the threshold to distinguish Al-accumulators [18, 19]. The dotted line indicates the critical K deficiency concentration [20]

For the calcifuge *A. flabellulatum*, Ca, Mg, Fe and Al concentrations in leaves and roots were higher but K concentrations in leaves and roots were lower when grown on the calcareous soil than on the acidic soil (*P* < 0.05, Fig. 3). When grown on the mixed soil, root Ca, Mg, Fe and Al concentrations of *A. flabellulatum* were higher and leaf K was lower than those on the acidic soil (*P* < 0.05).

In the calcicole *A. capillus-veneris* f. *dissectum*, root Ca, Mg, Fe and Al concentrations on acidic and mixed soils were lower and root K concentrations were higher than those on calcareous soil (*P* < 0.05, Fig. 3). Leaf Ca, Fe and Al concentrations, however, were higher and leaf P and K were lower when grown on the acidic soil than on the calcareous soil (*P* < 0.05). When grown on the mixed soil, all leaf element concentrations were similar to those on the calcareous soil (*P* > 0.05).

Root Ca, Mg and P, and leaf Mg and P concentrations of *A. malesianum* were lower when grown on the acidic soil than on the calcareous soil (*P* < 0.05, Fig. 3). When grown on the mixed soil, root Fe and leaf P concentrations of *A. malesianum*, relative to those grown on the calcareous soil, were higher, but leaf Ca, Fe, Al and root P concentrations were lower (*P* < 0.05).

When grown on the acidic soil, *A. flabellulatum* had higher leaf K concentration, but lower leaf and root Ca, Fe, Al concentrations than the other two species (*P* < 0.05, Fig. 3). When grown on the calcareous and mixed soils, however, leaf K and P of *A. flabellulatum* were lowest. Relative to *A. malesianum*, *A. capillus-veneris* f. *dissectum* exhibited higher leaf Mg but lower leaf Al concentration when grown on the same soil (*P* < 0.05), and lower leaf Ca and Fe but higher root Ca, Mg, Fe and Al when grown on the calcareous soil (*P* < 0.05).

### Correlations between leaf element and plant growth

Final total biomass and RGR of the calcifuge *A. flabellulatum* correlated positively with leaf K and negatively with leaf Mg, and RGR correlated negatively with leaf Ca (*P* < 0.05, Table 2). For the calcicole *A. capillus-veneris* f. *dissectum*, SPAD and RGR correlated negatively with leaf Ca, Fe and Al, but positively with leaf P and K (*P* < 0.05). For the calcicole *A. malesianum*, positive correlation coefficients were found for final total biomass and RGR with leaf P and K, and final total biomass and SPAD with leaf Mg (*P* < 0.05).

**Table 2:** Pearson correlation coefficients between leaf element concentrations and final total biomass, SPAD values and relative growth rate (RGR) for three *Adiantum* species

	Leaf element concentration (mg g^− 1^)					
	Ca	Mg	Fe	Al	P	K
*A. flabellulatum*						
Final total biomass (g)	−0.43	− 0.48^*^	− 0.34	− 0.35	− 0.37	0.53^*^
SPAD	− 0.16	− 0.24	− 0.32	− 0.36	− 0.27	0.40
RGR (mg g^− 1^ d^− 1^)	− 0.48^*^	− 0.55^*^	−0.38	− 0.40	−0.40	0.67^**^
*A. capillus-veneris* f. *dissectum*						
Final total biomass (g)	−0.42	0.07	−0.27	−0.29	0.16	0.09
SPAD	−0.67^**^	−0.12	− 0.67^**^	−0.51^*^	0.66^**^	0.66^**^
RGR (mg g^−1^ d^− 1^)	− 0.67^**^	0.10	− 0.67^**^	− 0.52^*^	0.66^**^	0.49^*^
*A. malesianum*						
Final total biomass (g)	−0.43	0.52^*^	−0.28	−0.36	0.50^*^	0.53^*^
SPAD	−0.03	0.67^**^	0.12	−0.32	0.33	0.32
RGR (mg g^−1^ d^−1^)	−0.41	0.43	−0.32	−0.35	0.71^**^	0.63^**^

^*, **^ indicate coefficient significant at *P* < 0.05 and 0.01, respectively (*n* = 21)

## Discussion

Compared with acidic soils, calcareous soils are rich in Ca and Mg and have a neutral or higher pH [1, 3]. Some calcifuge plants adapted to severely Ca-impoverished soils do not strongly down-regulate their Ca uptake capacity, and, consequently, are highly sensitive to calcareous soils [9]. They are excluded from calcareous soils also by their lacking ability to fulfill their requirements for other essential elements [6, 8], and this limitation may be further exacerbated by the reduced element availability at alkaline pH [21]. In contrast, calcicole plants can tolerate excess Ca and take up nutrients when grown on calcareous soils [6]. In the present study, the pH, Ca, Mg and P concentrations of the calcareous soil were higher but K concentration was lower than those of the acidic soil (Table 1). After 120 d of growth, all three *Adiantum* species on the calcareous soil exhibited higher root Ca and Mg concentrations than those on the acidic soil (Fig. 3), but their leaf Ca, Mg, P and K, and RGR responded differently to soil types. When cultivated on the calcareous soil, leaf Ca and Mg concentrations of the calcifuge *A. flabellulatum* increased and leaf K, SPAD and RGR decreased (Figs. 2 and 3). The Ca and Mg requirements considered adequate for crop growth are 5.0 mg Ca g^− 1^ and 2.0 mg Mg g^− 1^ in leaves, respectively [16]. Leaf K concentration of 8.0 mg g^− 1^ was suggested by De Wit et al. [20] based on Dutch agricultural grasslands as a tentative indicator of K deficiency. Leaf Ca, Mg and K of *A. flabellulatum* were correlated with RGR (Table 2), but the former two elements on the calcareous soil were much more than the critical adequate concentrations (Fig. 3a, b) and leaf K was less than the critical deficiency value (Fig. 3f). In general, leaf Mg concentration beyond the growth-limiting level is unlikely to be detrimental because it can be stored mainly in the vacuoles, as a buffer for Mg^2+^ homeostasis in the metabolic pool and for charge compensation and osmoregulation in the vacuole [17]. In contrast, the strict compartmentation of Ca^2+^ at cellular level and maintenance of low Ca^2+^ concentrations in the cytosol are less effective in calcifuges than in calcicoles [17]. Therefore, K deficiency and excess Ca may exclude the calcifuge *A. flabellulatum* from calcareous soils. Conversely, leaf Ca concentration of the calcicole species on the calcareous soil did not change for *A. malesianum* and decreased for *A. capillus-veneris* f. *dissectum*, and leaf P and K concentrations, SPAD and RGR increased when compared with those grown on the acidic soil (Figs. 2 and 3), suggesting they can thrive on the calcareous soil by increasing leaf P and K uptake and retaining Ca in roots.

In the karst regions of southern China, limestone soils (calcareous) and red soils (acidic) are interlaced [1, 3]. Hence, they may “pollute” each other when extreme precipitation events occur [5]. In this study, we mixed the calcareous soil and the acidic soil at 1:1 and found the mixed soil had higher pH, Ca, Mg and P concentrations and lower K concentration than the acidic soil (Table 1). When grown on the mixed soil, the calcifuge *A. flabellulatum*, relative to its preferred acidic soil, had higher root Ca and Mg concentrations and lower leaf K, SPAD and RGR (Figs. 2 and 3). Such results showed *A. flabellulatum* will grow worse if the acidic soil is “polluted” by calcareous soils. For the calcicole *A. capillus-veneris* f. *dissectum*, leaf element concentrations and RGR were similar between the mixed soil and the calcareous soil (Figs. 2 and 3), indicating its growth will remain almost unaffected when the calcareous soil is “polluted” by acidic soils. On the contrary, the calcicole *A. malesianum* grew better, as evidenced by higher RGR (Fig. 2b), when cultivated on the mixed soil than on the calcareous soil. Chrysargyris et al. [22] suggested well balanced nutrients can improve plant growth by affecting the availability, transport, and partitioning of the nutrients. We also thought the element levels of the mixed soil, although lower P concentration relative to the calcareous soil (Table 1), might be more balanced for *A. malesianum*, and hence its RGR were highest and the leaf P was close to the sufficient concentration of 2.0 mg g^− 1^ considered adequate for crop growth [16] (Fig. 3e). In addition, the higher leaf P for *A. malesianum* on the mixed soil, compared to the calcareous soil, could be also related to lower soil Ca and Mg concentrations. In soils, Ca and Mg can reduce P bioavailability by forming poorly soluble Ca-P and Mg-P [23–25].

Iron deficiency is also suggested to exclude some calcifuge plants from calcareous soils [6] and Al toxicity excludes calcicole plants from acidic sites [12]. In this study, Fe and Al were similar among acidic, calcareous and mixed soils (Table 1). The calcifuge *A. flabellulatum*, however, had higher leaf and root Fe concentrations and its leaf Fe exceeded the critical toxicity concentration of 0.5 mg g^− 1^ [17] when grown on the calcareous and mixed soils than on the acidic soil (Fig. 3c). Therefore, its chlorotic and necrotic symptoms on leaves when forced to grow on calcareous or mixed soils might not be due to a lack of Fe. However, we only measured total leaf Fe and not biologically available Fe. Zohlen and Tyler [8] argued Fe immobilization in physiologically less active forms in leaf tissue may also exclude calcifuge plants with adequate total leaf Fe from calcareous soils. In general, a value of 1.0 mg Al g^− 1^ leaf dry mass is a threshold to distinguish Al accumulators and non-Al accumulators [18, 19]. The calcifuge *A. flabellulatum* and the calcicole *A. capillus-veneris* f. *dissectum* might be Al excluder species because their leaf Al concentrations were lower than the threshold when grown on their respective preferred soils (Fig. 3d), whereas the calcicole *A. malesianum* might be an Al includer species since its leaf Al concentrations were more than the threshold on all three soil types (Fig. 3c, d). When forced to grow on the acidic soil, *A. capillus-veneris* f. *dissectum* had lower RGR (Fig. 2b) and its leaf Al concentration exceeded the threshold (Fig .3d). The results, however, might not indicate the calcicole species experienced Al toxicity. First, the evident symptoms of Al toxicity, which the root apices and laterals became thick, stubby and brown in appearance [26], did not occur. Second, all the Ca/Al molar ratios in roots, the superior indicators than the leaf Al concentration for evaluating Al toxicity and acidity stress to plants [26, 27], were above the critical 0.2 for the calcicole species on three soil types.

Wang et al. [2] found that *A. capillus-veneris* f. *dissectum* was a calcicole species with low Ca concentration. In our study, leaf Ca concentration of *A. capillus-veneris* f. *dissectum* was similar to the calcifuge *A. flabellulatum* but lower than the calcicole *A. malesianum* when grown on their respective preferred soils (Fig. 3a). The results further indicated that the calcicole *A. capillus-veneris* f. *dissectum* is a low leaf Ca species. Nevertheless, its root Ca concentration was highest among the three species (Fig. 3a), implying a Ca exclusion strategy enabling it to avoid excess leaf Ca by retaining Ca in roots. In contrast, the calcicole *A. malesianum* had an almost 2-fold greater leaf Ca concentration than *A. capillus-veneris* f. *dissectum* under calcareous conditions (Fig. 3a), suggesting it take up the extra Ca and can tolerate the higher leaf Ca. The tolerance is most likely achieved through biomineralization of excess Ca, forming Ca-based minerals (presumably Ca-oxalate), thus avoiding any interference from Ca^2+^ on cell functioning and the availability/allocation of other nutrients [28]. This idea of interspecific variation in the ability of *Adiantum* species to form Ca-oxalate crystals is supported by Anthoons [29].

## Conclusion

The calcifuge *A. flabellulatum* performed worse on the calcareous soil. Such a response might be attributed to the increased leaf Ca concentration and decreased leaf K concentration. In contrast, the calcicole *A. capillus-veneris* f. *dissectum* and *A. malesianum* could effectively take up P and K to leaves and hence thrive on the calcareous soils. Relative to *A. malesianum*, *A. capillus-veneris* f. *dissectum* is a low leaf Ca calcicole species. If their preferred calcareous soils are “polluted” by acidic soils, *A. capillus-veneris* f. *dissectum* can remain almost unaffected while *A. malesianum* will grow better. Conversely, *A. flabellulatum* will grow worse if its optimum acidic soil is “polluted” by calcareous soils.

## Methods

### Plant materials and growth conditions

*A. capillus-veneris* f. *dissectum* (M. Martens & Galeotti) Ching, *A. malesianum* J. Ghatak and *A. flabellulatum* L. are perennial evergreen ferns of the family Pteridaceae. In the karst region of Guangxi, South China, *A. capillus-veneris* f. *dissectum* and *A. malesianum* occur on limestone soils (calcareous), but *A. flabellulatum* is restricted to red and yellow soils (acidic). *A. capillus-veneris* f. *dissectum*, height 15–40 cm, features slender and creeping rhizomes, and ovate-triangular and 1- or 2-pinnate laminas. *A. malesianum*, 10–40 cm tall, possesses short and erect rhizomes and whiplike 1-pinnate laminas, and can form new plantlet by rachis rooting at apex. *A. flabellulatum*, named after its flabellate and 2- or 3-dichotomously branched laminas, is 20–45 cm tall and possesses short and erect rhizomes.

These three *Adiantum* species can be propagated by rhizome separation. In April 2017, mother plants of *A. capillus-veneris* f. *dissectum* and *A. malesianum* were obtained from Yangshuo County, Guangxi (24°43′N, 110°30′E), and *A. flabellulatum* were collected from Guilin Botanical Garden, Guangxi, South China (25°04′N, 110°18′E). They were identified by Professor Yan Liu from Guangxi Institute of Botany and Voucher specimens were deposited at the Guangxi herbarium (http://www.gxib.cn/spIBK). For each species, 7 mother plants with similar size were selected and each plant was separated into three cloned individuals. The three clones were randomly assigned to three pots (height 16 cm and diameter 18 cm) containing different soil types (calcareous, acidic and mixed soils, 2 kg). Thus, there were 21 pots (7 individuals × 3 soil types) for each species. The pots stayed on benches, randomly distributed inside a shaded plastic greenhouse, according to Liao et al. [15]. During the period (April 28 to August 26, 2017), the average day/night temperature and humidity were 30.4/23.3 °C and 75.1%, respectively.

The calcareous soil was collected from root zone (the top 20 cm of soil within a 20 cm radius) of *A. capillus-veneris* f. *dissectum* from Yangshuo County, Guangxi, and the acidic soil was obtained from *A. flabellulatum* root zone in Guilin Botanical Garden, Guangxi, South China. These soils were dried in the shade under room temperature and sieved (0.5 cm) to remove pebbles, leaves and unwanted material. The mixed soil was a mixture of the calcareous and the acidic soils (1:1, v/v). For each pot, soil was watered to 60% of field capacity every 2 days by commonly-used weight method.

### Measurements

Before being potted, soil pH was determined in CaCl_2_, and total Ca, Mg, Fe, Al, P, K concentrations were determined by inductively coupled plasma–optical emission spectrometry (ICP-OES, Agilent 725, Agilent Technologies, USA) after microwave digestion with 1:3 HNO_3_:HCl [30]. The initial fresh weights of all plant individuals were recorded, and their dry weights (initial total biomass, DW_1_) were estimated based on the dry weight/fresh weight ratios of subsamples analyzed in the laboratory.

After 4 months of growth, chlorophyll levels of newly formed and mature leaves were determined using a SPAD-502 chlorophyll meter (Minolta, Osaka, Japan) and then all the plants were harvested. For each individual, final total biomass (DW_2_) was obtained after being washed with tap water carefully and then oven-dried at 80 °C for at least 24 h. From DW_1_ and DW_2_, the relative growth rate per day (RGR, mg g^− 1^ d^− 1^) was estimated as (ln DW_2_ – ln DW_1_)/120 × 1000. After measuring the biomass, leaf and root samples were ground and homogenized. The samples were digested with 5:1 HNO_3_:HClO_4_ [13] and the concentrations of Ca, Mg, Fe, Al, P and K were determined by inductively coupled plasma-mass spectrometry (ICP-MS, iCAP-Qc, ThermoFisher Scientific, USA).

### Statistical analysis

Differences of soil properties were compared using one-way analysis of variance (ANOVA) followed by least significant difference (LSD) (*P* < 0.05). Differences of SPAD values, element concentrations (Ca, Mg, Fe, Al, P and K) among species and soil types were tested by two-way ANOVA. Since biomass production and RGR are initial size dependent [31], their differences among species and soil types were tested by two-way analysis of covariance (ANCOVA), with DW_1_ as a covariate. For each species, linear correlations between the measured leaf element concentrations and final total biomass, SPAD values and RGR were tested by Pearson correlation coefficient. All analyses were conducted using SPSS 20.0 for Windows (SPSS Inc., Chicago, USA).

## Data Availability

The datasets used and/or analysed during the current study are available from the corresponding author on reasonable request.

## Abbreviations

*Af*: *Adiantum flabellulatum*
*Ac*: *Adiantum capillus-veneris* f. *dissectum*
*Am*: *Adiantum malesianum*
RGR: Relative growth rate
DW_1_: Initial total biomass
DW_2_: Final total biomass
